# Factors affecting women scientists’ retention and progress in STEM fields in the UAE: A cross-sectional study

**DOI:** 10.12688/f1000research.155420.1

**Published:** 2024-12-20

**Authors:** Azhar T. Rahma, Javaid Nauman, Alia Albawardi, Hajer Alyammahi, Rim Fares, Payaswini Saikia, Aminu S. Abdullahi, Abubaker Suliman, Linda Zou, Saeeda Almarzooqi

**Affiliations:** 1 Institute of Public Health, College of Medicine and Health Sciences, United Arab Emirates University, Al Ain, Abu Dhabi, 15551, United Arab Emirates; 2 Department of Circulation and Medical Imaging, Faculty of Medicine and Health Sciences, Norwegian University of Science and Technology, Trondheim, Norway; 3 Pathology, College of Medicine and Health Sciences, United Arab Emirates University, Al Ain, Abu Dhabi, United Arab Emirates; 4 Khalifa University, Abu Dhabi, Abu Dhabi, United Arab Emirates; 5 Department of Physics, College of Science, United Arab Emirates University, Al Ain, Abu Dhabi, 15551, United Arab Emirates; 6 Center for Astrophysics and Space Science, New York University, Abu Dhabi, 129188, United Arab Emirates

**Keywords:** STEM, women retension, scientists in STEM, gender, retention, qualitative research, professional practice, attrition, inclusive and engaging environments, induction and retention

## Abstract

**Background:**

The representation of women in science, technology, engineering, and mathematics (STEM) is disproportionate to graduates from STEM fields. There is limited research addressing challenges facing women retention in STEM in the UAE.

**Methods:**

A cross-sectional study using a validated questionnaire was conducted. A total of 165 participants were enrolled; 62% males and 35% females.

**Results:**

More women believed there is gender inequality in STEM (47% versus 28%). 44% of female participants experienced gender inequality in their career. Men were significantly less likely to experience gender inequality (OR=0.06, 95% CI=0.02-0.16). Women reported lack of organizational emphasis on diversity and inclusion for promotion to leadership (44% versus 60%).

**Conclusion:**

Data confirms gender-based preconceptions and biases in STEM fields. Institutional initiatives and policies to challenge stereotypes and promote gender equality are required. Governmental role is crucial in creating an inclusive environment for women scientists.

## Introduction

The representation of women working in professions related to science, technology, engineering, and mathematics (STEM) is disproportionate to the percentages of those graduating from STEM fields. Worldwide, multiple studies documented an increase in the number of women receiving postgraduate degrees but a relatively static representation of women in faculty positions in STEM fields ( Casad et al. 2022; Roper 2019). The World Economic Forum reports a persistent gender gap in STEM fields; for example, women graduate in information and communication technology represent 1.7% compared to 8.2% of men graduates. Similarly, women represent only 6.6% of engineering and manufacturing graduates compared to 24.6% for men ( “World Economic Forum” 2022).

A study from MIT published in 1999 showed that senior women faculty felt discrimination in salary, awards and resources in spite of the same qualification and competency. Subsequently, the MIT initiated recommendations to increase representations of women in STEM. Despite their efforts, the increase in recruitments were not sustainable after few years ( Lawler 2006).

Women in the United Arab Emirates (UAE) constitute around 2/3 of students enrolled at federal higher education institutions and around half at private institutions ( “UAE GBC Women Facts” 2022). Furthermore, women represent 41.49% of STEM field graduates in the UAE ( “World Economic Forum” 2022). However, representations in the workforce is still disproportionate ( Patterson, Varadarajan, and Salim 2021). Employment rate is lower for women in comparison to men in computer information sciences and engineering according to a study looking at graduates in 2015-16 at higher colleges of technology and the UAE university ( Houjeir et al. 2019).

The disparity is postulated to be related to gender stereotyping impacting recruitment and career advancement, limited social networks and existing work climates in academia ( Casad et al. 2022). It was also shown that social exclusion from men dominated fields resulted in fewer career opportunities for women ( Cyr et al. 2021). In the UAE, similar social and gender factors play a role in the lower representation of women in workforce in STEM fields ( Houjeir et al. 2019).

Two recent review articles addressing women in STEM in the UAE highlighted some of the challenges in the field (6 and 9). In one review, it was shown that male-dominance in fields like engineering and difficulty in having a clear promotion track resulted in women to leave engineering and pursuing other fields of work ( Alzaabi, Ramirez-Garcia, and Moyano 2021). Social factors and family demand on women in addition to some societal gender-biases are attributable to lower number of women working in STEM ( Patterson, Varadarajan, and Salim 2021).

In the US, the National Institute of Health (NIH) and the National Science Foundation (NSF) supported grants to investigate gender disparity in STEM careers. One notable initiative is the NSF-funded ADVANCE interventions ( Casad et al. 2022). It included interventions to enhance recruitment of women in STEM, improve academic climate and develop mentoring and networking ( Casad et al. 2022). Higher education institutions can benefit from the evidence based research work and the proposed StratEGIC Toolkit developed by some investigators to enhance women representation in STEM. Using this toolkit, institutions can implement structural changes that support women’s advancement in STEM fields based on evidence-based guidelines ( Laursen and Austin 2014). In a recent study, investigators used comics and text-only tweets to increase awareness about underrepresentation and stereotypical biases about women in STEM ( Freedman et al. 2022).

In the UAE, multiple government initiatives are in place to encourage students to enroll to STEM fields. However, there is limited research looking into challenges facing women working in STEM fields in the UAE ( Patterson, Varadarajan, and Salim 2021).

In the current study, we aim at exploring these challenges to better understand the current situation. This will help in providing recommendations that allow better retention of women in STEM fields.

## Methods

To achieve the aim of this research, a cross-sectional study design was adopted. A validated questionnaire was used to explore and assess the factors affecting women scientists’ retention and progress in STEM fields in the UAE ( Rentsch and Steel 1992; “ Canadian Nuclear Safety Commission (CNSC) Annual Report 2019–20”; Hopkins 2002). The questionnaire (consisting of 28 open and closed-ended questions - see extended data 1) was divided into three sections: demographic, attitudes questions, and the Andrews and Withey job satisfaction questionnaire. The questionnaire was piloted from August-September 2022 and Cronbach alpha had been calculated and questions with low score (less than 0.7) were removed.

### Study population

The study included men and women aged 18 years or older who were graduating or working in STEM fields (science, technology, engineering, and mathematics) in the UAE.

### Sample size calculation

To determine the sample size, the WHO calculator for cross-sectional studies was used with the following variables:

Level of Confidence Measure: 1.96 (for 95% confidence level)

Margin of Error (MOE): 0.05

Baseline levels of the indicators: 0.5

Design effect (Deff ): 1

Expected Response Rate: 0.8

### Sampling

The sampling frame was from academia and research institutions in the UAE, as well as institutions with STEM fields like hospitals, government entities, and schools. All the mentioned locations were contacted, and participants were asked to sign a consent form before taking the survey. Snowball sampling was also used to distribute the survey via social media (WhatsApp groups). The distribution period was from November 2022 till July 2023.

### Outcome and exposure variables

The main outcome studied was “Factors Affecting Women Scientists’ Retention and Progress in STEM fields in the UAE.” The studied exposure variables included age, gender, marital status, nationality, education level, employment status, and family structure.

### Measurements and Analysis

R software version 4.1.2. ( Chen et al. 2023) was used to analyze the data. Variables were summarized and presented as frequencies and percentages. Univariate and multivariable binary logistic regression were used to explore the association between selected demographic factors – including age, gender, education level, and employer – with experience of gender inequality. Adjustments in the regression models were made for all independent variables to address potential confounding. Crude (cOR) and adjusted odds ratios (aOR) and their corresponding 95% confidence intervals were reported.

## Results

### Participants’ characteristics

A total of 165 participants (comprising 15% UAE nationals) took part in the survey with majority being males (62%). Most of the participants were between the age of 40-49 years (35%) ( Table 1). The majority (77%) were married with more than half (53%) of the participants reporting having 1-3 children under the age of 18. Most of the respondents had a doctorate (76%), were employed (92%), work in academic establishments (78%), and/or had more than 20 years of work experience (44%). Finally, 82% of the participants claimed to be the main bread-earners of their respective families.

**Table 1.** Characteristics of participants (N=165).

Characteristic	All, N (%)	Females, N (%)	Males, N (%)
**Gender**			
Female	62 (38%)	-	-
Male	103 (62%)	-	-
**Age**			
20-29 years old	8 (4.8%)	8 (13%)	0 (0%)
30-39 years old	37 (22%)	18 (29%)	19 (18%)
40-49 years old	57 (35%)	24 (39%)	33 (32%)
50-59 years old	39 (24%)	10 (16%)	29 (28%)
60-64 years old	9 (5.5%)	1 (1.6%)	8 (7.8%)
> 65 years old	15 (9.1%)	1 (1.6%)	14 (14%)
**Marital status**			
Married	127 (77%)	31 (50%)	96 (93%)
Unmarried	38 (23%)	32 (50%)	7 (7%)
**Highest education**			
Bachelor’s degree	10 (6.1%)	8 (13%)	2 (1.9%)
Master’s degree	30 (18%)	19 (31%)	11 (11%)
Doctorate degree	125 (76%)	35 (56%)	90 (87%)
**Nationality**			
UAE	24 (15%)	15 (25%)	9 (9.3%)
Other asians	58 (37%)	24 (40%)	34 (35%)
Africa	20 (13%)	7 (12%)	13 (13%)
America/Europe/Australia	55 (35%)	14 (23%)	41 (42%)
**Employment status**			
Employed	151 (92%)	49 (79%)	102 (99%)
Unemployed	14 (8.5%)	13 (21%)	1 (1.0%)
**Employer**			
College or university	127 (78%)	37 (62%)	90 (87%)
Federal, or government setting	20 (12%)	10 (17%)	10 (10%)
Others	16 (10%)	15 (21%)	3 (3%)
**Years of employment in STEM**			
<5 years	17 (10%)	13 (21%)	4 (3.9%)
5-10 years	28 (17%)	16 (26%)	12 (12%)
11-15 years	28 (17%)	13 (21%)	15 (15%)
16-20 years	19 (12%)	8 (13%)	11 (11%)
>20 years	73 (44%)	12 (19%)	61 (59%)
**Home main income source**			
Self	130 (82%)	36 (60%)	94 (96%)
Others	28 (18%)	24 (40%)	4 (4%)

### Factors influencing women to be in STEM

**Figure 1.**
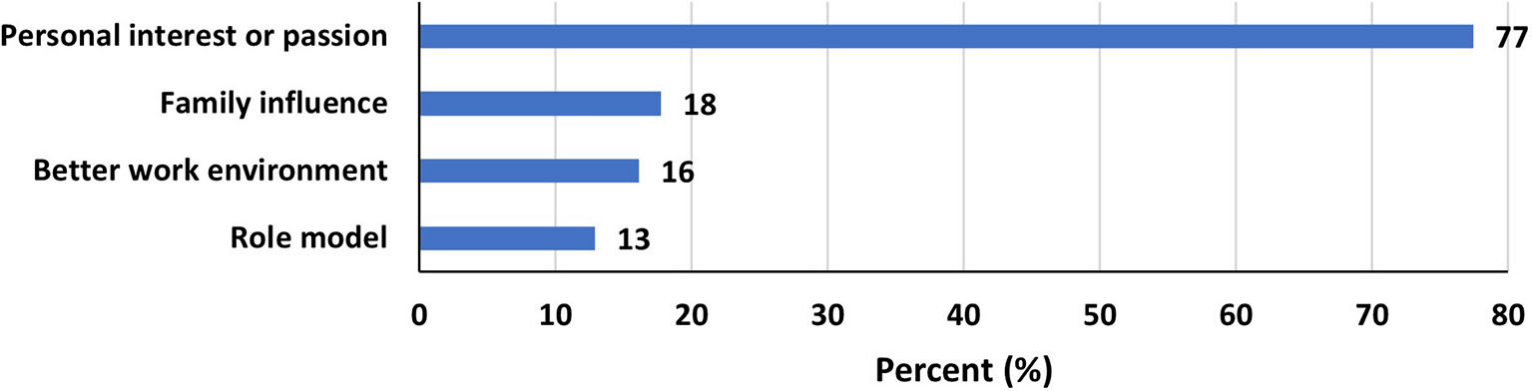
Most influencing factors to be in STEM among women.

Most women (77%) were influenced to join STEM driven by a personal interest or passion ( Figure 1). 18% of respondents were influenced by family and 16% chose a STEM field for a better work environment. Role models in STEM only influenced the choice in 13% of respondents.

### Feeling about current STEM job

**Figure 2.**
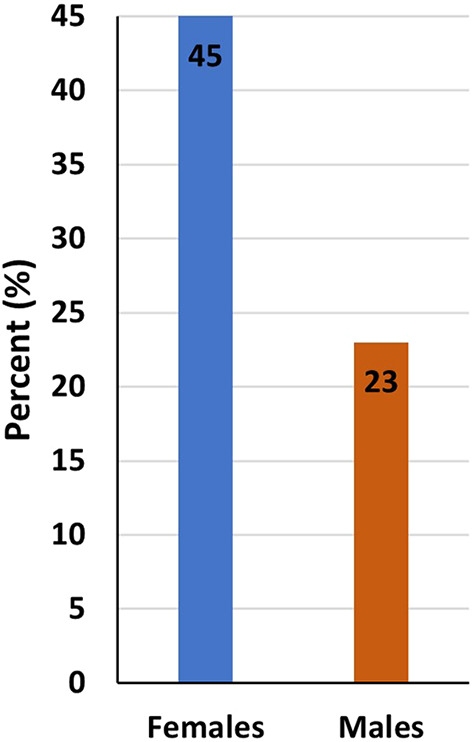
Considered quitting STEM job in the last 2-3 years.

**Figure 3.**
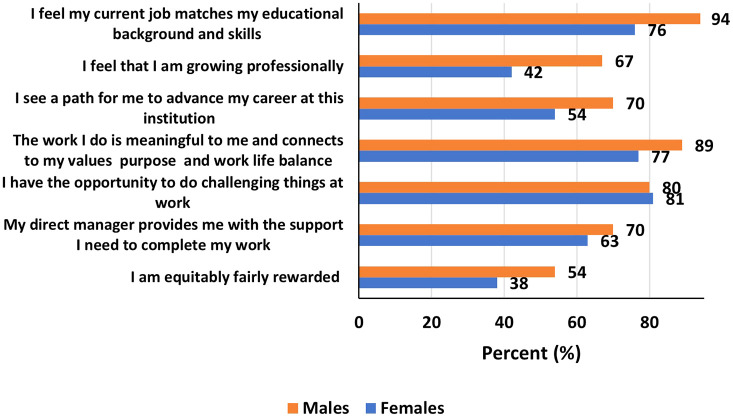
How participants feel about their current STEM job by gender.

Generally, a higher percentage of women (45% vs 23% for men) considered quitting their current STEM job in the last 2-3 years ( Figure 2). Overall, men were most likely to feel better in their current STEM job than women ( Figure 3). Specifically, more men, than women, reported that “they feel their current job matches their educational background and skills” (94% versus 76%), that “they feel they were growing professionally” (67% versus 42%), that “they see a path for to advance their career at their institution” (70% versus 54%), and that “they were equitably fairly rewarded” (54% versus 38%) among others.

### Gender inequality and the participants’ experience

**Figure 4.**
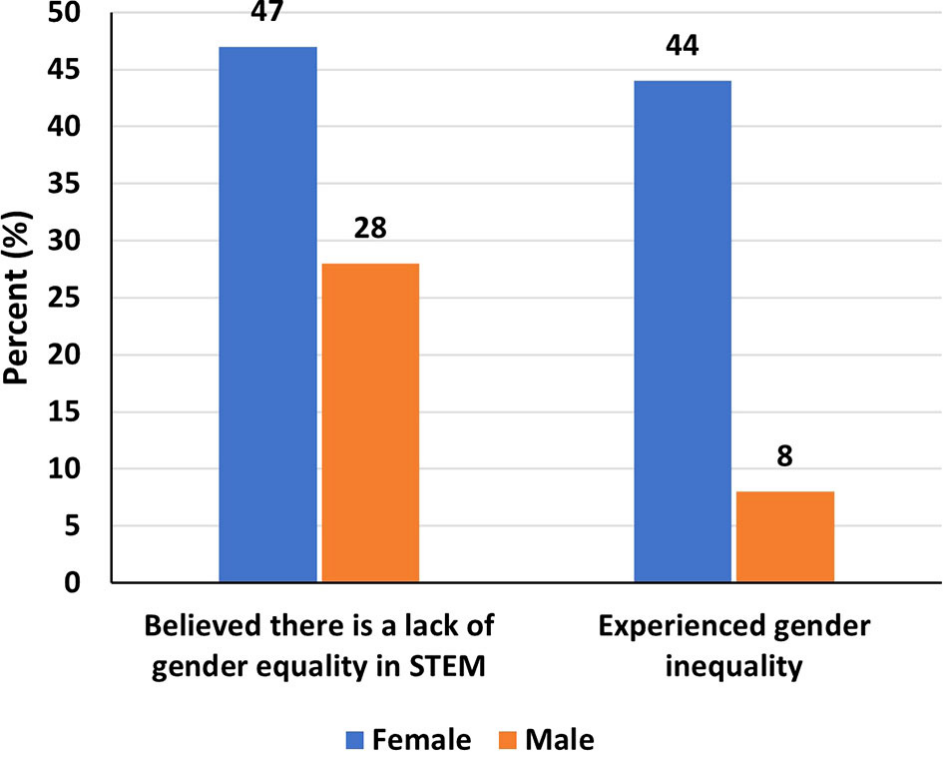
Gender inequality and the experience of women and men.

Of the respondents, 47% of women and 28% of men believed that there is a lack of gender equality in STEM ( Figure 4). Moreover, a significantly higher proportion of women reported experiencing gender inequality (44% women compared to 8% men). Females (aOR=15.8, 95% CI=6.08-45.7, p<0.001) and those in academic sector (aOR=3.38, 95% CI=1.15-11.3, p=0.035) were significantly more likely to experience gender inequality ( Table 2).

**Table 2.** Association between demographics and gender inequality experience.

Factors	cOR	95% CI	p-value	aOR	95% CI	p-value
**Age**						
< 40	1.00			1.00		
≥ 40	1.11	0.49, 2.70	0.800	2.00	0.75, 5.71	0.200
**Gender**						
Male	1.00			1.00		
Female	**9.16**	**3.96, 23.4**	**<0.001**	**15.8**	**6.08, 45.7**	**<0.001**
**Education**						
Bachelor’s degree	1.00			1.00		
Postgraduate	1.08	0.26, 7.40	>0.900	1.45	0.26, 11.4	0.700
**Employer**						
Others	1.00			1.00		
College or university	1.41	0.56, 4.06	0.500	**3.38**	**1.15, 11.3**	**0.035**

### Attitude towards challenges facing women in STEM

**Figure 5.**
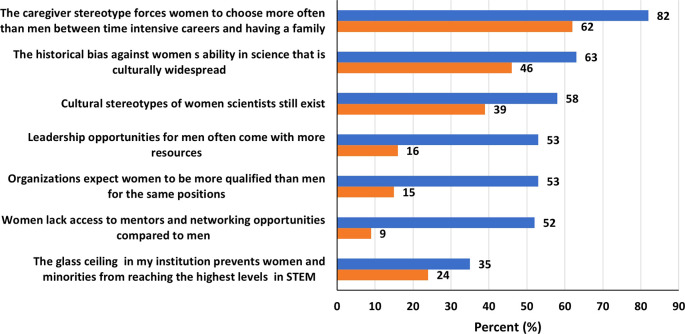
Attitude toward challenges facing women in STEM in the UAE.

Overall, women expressed negative attitudes regarding challenges facing them in STEM ( Figure 5). Majority of both women (82%) and men (62%) believed that “the caregiver stereotype forces women to choose more often than men between time intensive careers and having a family.” Additionally, majority of women also believed that “the historical bias against women’s ability in science that is culturally widespread” (63%), that “cultural stereotypes of women scientists still exist” (58%), that “leadership opportunities for men often come with more resources” (53%), that “organizations expect women to be more qualified than men for the same positions” (53%), and that “women lack access to mentors and networking opportunities compared to men” (52%). Furthermore, 35% of the women believed that “the glass ceiling in their institutions prevents women and minorities from reaching the highest levels in STEM.” In contrast, only a minority of men (16%) believed “leadership opportunities for men often come with more resources”, and only 15% believed that “organizations expect women to be more qualified than men for the same positions”, and only 9% of men believed that “women lack access to mentors and networking opportunities compared to men”. Lastly, a relatively comparable percentage of men (24%), in contrast to 35% women, believed a glass ceiling effect exist in STEM.

### Workplace challenges and experiences in STEM

**Figure 6.**
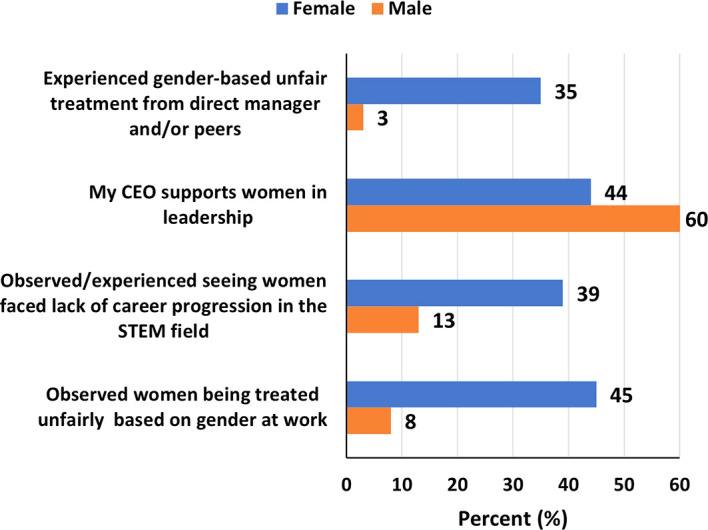
Workplace challenges and experiences faced by women and men in STEM in the UAE.

**Figure 7.**
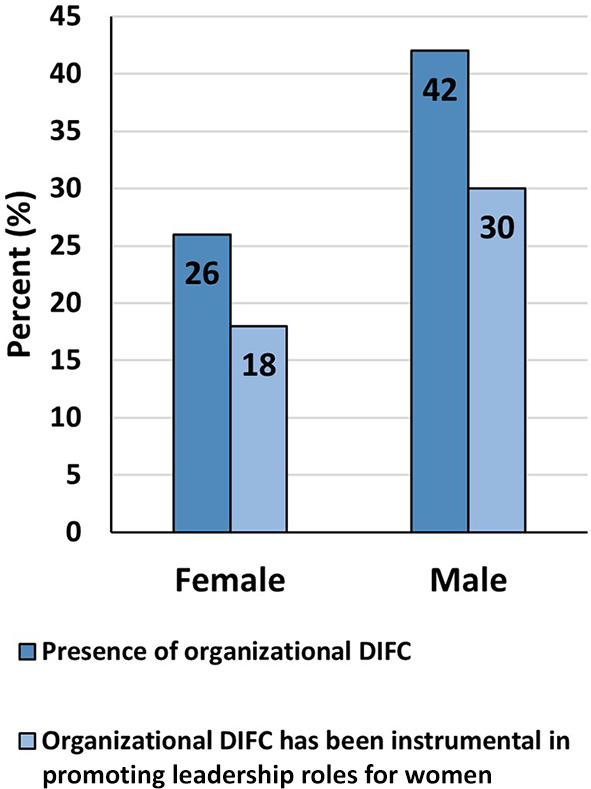
Presence and functioning of organizational diversity and inclusion-focused committee (DIFC) at participants’ workplaces.

More women, than men, reported having experienced gender-based unfair treatment from direct manager and/or peers (35% versus 3%), that they had observed/experienced seeing women face lack of career progression in the STEM field (39% versus 13%), and that they had observed women being treated unfairly at work based on gender (45% versus 8%). Forty-four percent and sixty percent of women and men respectively said that their CEOs supports women in leadership ( Figure 6). Although 42% of women and 30% of men said their organizations had diversity inclusion-focused committee (DIFC), only 26% and 18% respectively said the DIFCs had been instrumental in promoting leadership roles for women ( Figure 7).

### Support needed to thrive in STEM

**Figure 8.**
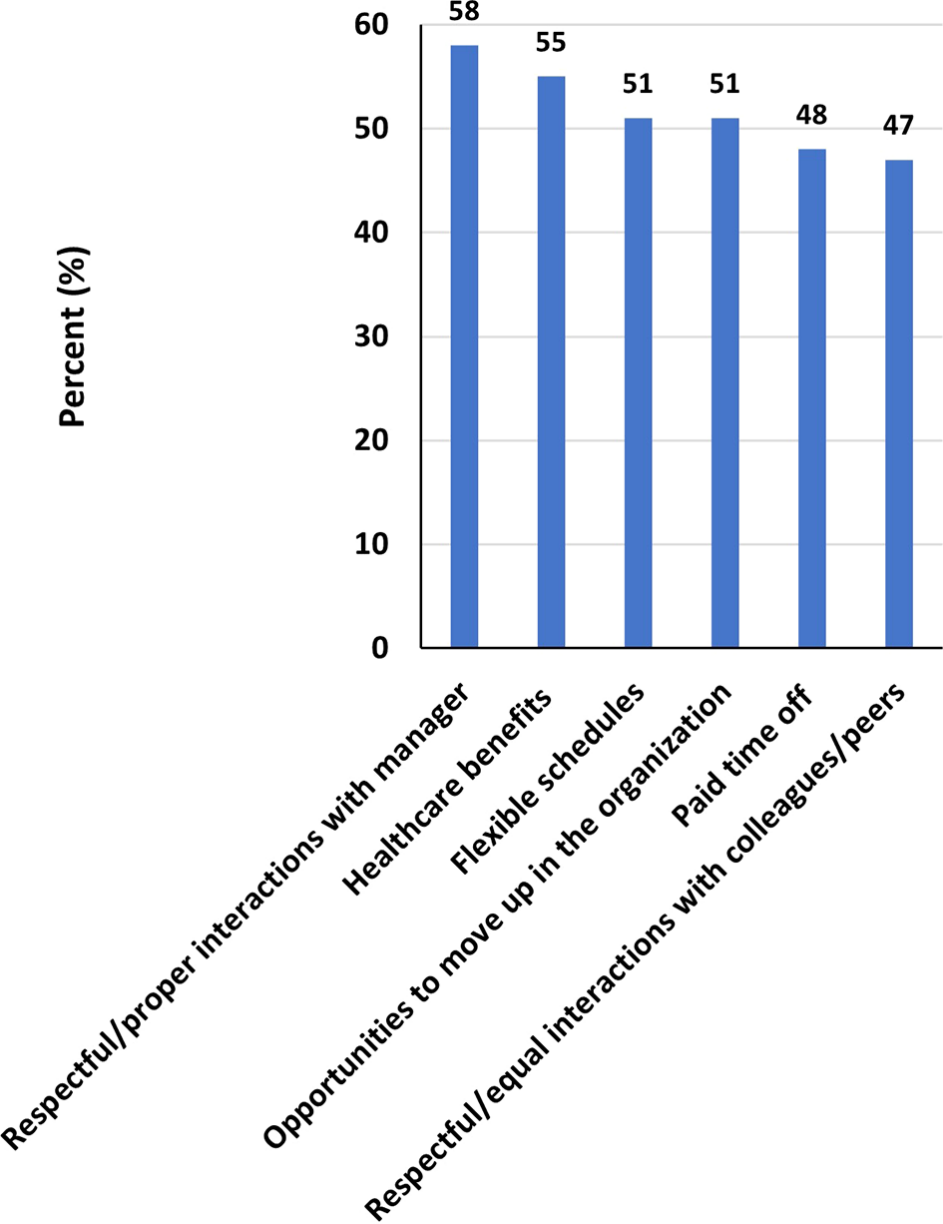
Support needed to thrive in STEM – Females.

**Figure 9.**
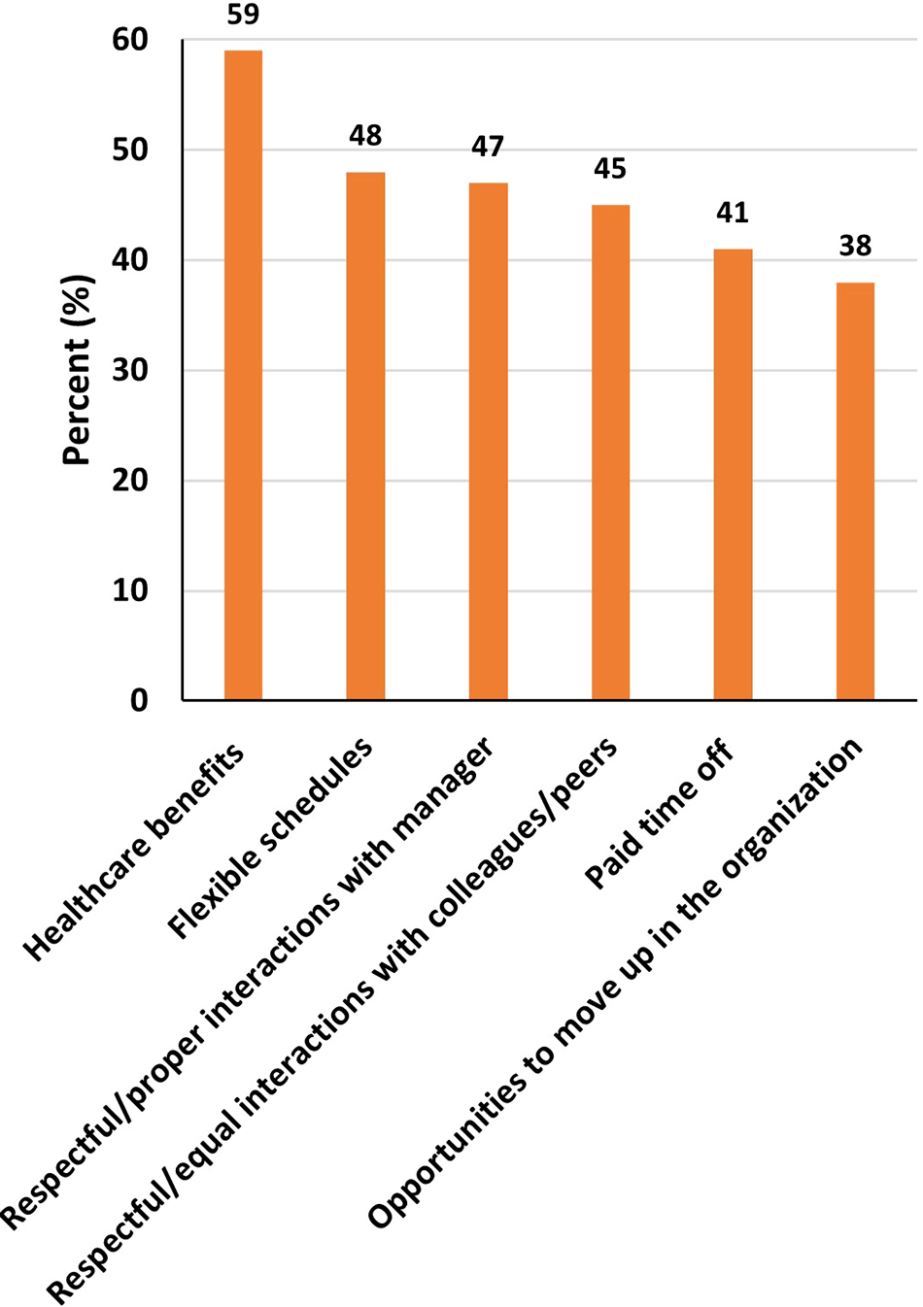
Support needed to thrive in STEM – Males.

Among women, the most common support needed to thrive in STEM were respectful/proper interactions with manager (58%), healthcare benefits (55%) and flexible schedules (48%) among others ( Figure 8). While for men, the most common support needed to thrive in STEM were healthcare benefits (59%), flexible schedules (48%), and respectful/proper interactions with manager (47%) among others ( Figure 9).

## Discussion

In this cross-sectional analysis of adult men and women who either graduated or were working in the STEM fields in multiple institutions of higher education across the United Arab Emirates, we found that more women reported to experience gender-based unfair treatment from direct managers and/or peers, lacked access to mentors and networking opportunities compared with their male counterparts, and organizations provided more resources to men for leadership opportunities while asking women to be more qualified for the same positions. Furthermore, more men than women reported to agree that their current job matched with their educational background and skills, and more women than men considered the possibility of quitting the job in STEM in the last 2-3 years. For retention in the STEM, women rated flexible work schedule, proper interactions with direct manager, and tenure promotional opportunities higher than men.

Our results are consistent with the findings of earlier studies demonstrating the prevalence of gender bias in science disciplines, and how this bias may affect the professional hiring, promotion, mentoring, and funding opportunities ( Moss-Racusin et al. 2012; Steinpreis, Anders, and Ritzke 1999; Reuben, Sapienza, and Zingales 2014) . The findings of a double blind RCT indicated that male applicants for a lab manager position, evaluated by both male and female science faculty, were significantly more likely to be hired, receive a higher annual salary, and to get more mentoring opportunities than the female applicants ( Moss-Racusin et al. 2012). Similar results were reported by others across diverse fields showing that job applicants with female names were less likely to be called by employers ( Quadlin 2018) , woman faculty member was also less likely to receive tenure because her research contributions are often undervalued ( Sarsons 2017), and male applicants were favored over females for peer review in grant fundings ( Robyn et al. 2018).

Approximately 40% of women in our study considered leaving STEM, and these results are in line with a recent publication showing comparable numbers ( Conrad, Abdallah, and Ross 2021). Many factors have been reported to affect the women retention in the STEM which include but not limited to work life balance or flexibility, unequal standards for men and women, stress and gender discrimination ( Conrad, Abdallah, and Ross 2021; Blackburn 2017; Smith and Gayles 2017). The women in our study rated flexible work schedule much higher than men to stay in their respective jobs. The lack of flexibility regarding family commitments has been recognized as a factor motivating women to pursue careers outside of STEM ( Conrad, Abdallah, and Ross 2021). Other factors rated higher by the women in our study were the support systems in terms of clear communications with direct managers and promotional opportunities. The results of a study of junior biomedical researchers comprising of 92 women and 127 men who applied for early-career grant fundings showed that junior faculty women received significantly less start-up support compared with men from their institutes ( Sege, Nykiel-Bub, and Selk 2015). The lack of these support systems could influence the persistence of women in their chosen careers ( Conrad, Abdallah, and Ross 2021).

There is also evidence that these specific challenges faced by women during their professional careers in STEM may even persist during the periods of college or university education, and can have career-impacting effects. Previous research has identified various factors, including low level of self-efficacy despite being equally prepared ( Koch et al. 2022), inadequate support from family, school and faculty ( Tandrayen-Ragoobur and Gokulsing 2021), as well as other psychosocial and non-cognitive elements ( Ortiz-Martínez et al. 2023) that might contribute to women showing lower persistence rates than men when it comes to completing a STEM degree. The findings also suggest that gender gap in STEM education is not an isolated issue but rather a systemic challenge.

Several strategies have been proposed to address the gender disparity in STEM areas using various approaches, including efforts related to attraction, access, and retention ( García-Peñalvo et al. 2019). The framework proposed by Eddy and Brownell ( Eddy and Brownell 2016) identified observable inequalities in performance and engagement as factors contributing to gender gaps. Makarem and Wang ( Makarem and Wang 2019) highlighted various coping strategies women generally employ, including conforming to expectations, engaging in impression management, and taking proactive steps to assert themselves and overcome obstacles, to counter the challenges related to predominantly male-dominated environments, including gendered organizational culture and stereotypes. Furthermore, the underrepresentation of women in STEM filed is partly due to systemic obstacles to the recruitment, retention and promotion, and institutes should consider implementing strategies to change the structures and climates of workplaces, and to create a more inclusive and supportive environment for women pursuing STEM careers ( DeAro, Bird, and Mitchell-Ryan 2019).

The international organizations have emphasized on critical significance of addressing the gender disparity in higher education, specifically within the STEM. The United Nations’ Sustainable Development Goal 4, with a specific focus on target 4.3, calls for equal access to tertiary education, including universities, for both women and men ( Heleta and Bagus 2020). Data from the Organization for Economic Co-operation and Development (OECD) show a significant improvement in the fields of natural sciences, mathematics, and statistics, achieving a state of gender parity. However, this achievement contrasts with the persistence of a gender gap in fields like engineering and information and communication technologies. The OECD also highlights the importance of eradicating stereotypes, implementing gender balance policies across various academic disciplines, and actively cultivating an inclusive environment to encourage greater female participation in traditionally male-dominated fields ( Organisation for Economic Co-operation and Development 2021; OECD 2021).

These findings are congruent with the observation that the majority of CEOs and members of higher management in STEM related institutions are men.

Women reported higher career challenges when it comes to socio-cultural roles, lifestyle values and work-family balance. Awareness-raising campaigns and providing flexible work arrangements can help women balance their work and family responsibilities.

Male participants strongly agreed that their current job matches their educational background and skills (80% vs 45%). This may be explained in view of bias in hiring or gender related personal traits and inclinations. More questions are needed to clarify reasons behind participants’ input. For example, some may feel that they are over-qualified for the current job or vice versa.

Interestingly, only 26% of women and 42% of men reported having an organizational diversity and inclusion focused committee. The latter committee when present, proved useful in ensuring a more inclusive and supportive environment for women. Thus, institutional responsibility towards employees and community is of paramount importance. Appropriate institutional policies and procedures help close the gender gap in STEM and ensure that women have the same opportunities as men to succeed in these fields.

Up to our knowledge, this study is the first to investigate retention and progression challenges encountered by women in STEM fields in the UAE. It examines this issue from the perspectives of both male and female individuals in STEM fields across various institutes within the UAE. Employing a validated questionnaire, ensures a systematic exploration of pertinent factors and allow for benchmarking with other countries in the globe. Furthermore, the piloting process and calculation of Cronbach’s alpha underscore the methodological rigor and reliability of the study. The study has some limitations. One is that it lacks data on the social inclusion of women in STEM, a critical aspect for comprehending broader socio-cultural influences on their experiences in the field. But this is important to know, since social factors can have a big impact on women’s experiences in STEM. For example, research has shown that stereotypes can make women feel less engaged and less like they fit in at work. So, not having information about social inclusion makes it harder to understand the full picture. Another limitation is that the study doesn’t look at differences between different types of STEM fields, like engineering and science. Research has shown that these fields can have different challenges and working environments. So, not accounting for these differences could make the findings less specific. We recommend conducting longitudinal research tracking the career trajectories of women in STEM fields over time to provide valuable insights into the factors influencing their career decisions and outcomes. Understanding how these factors evolve over time can inform strategies for improving retention and promoting gender diversity in STEM and can be insightful for policy makers.

## Conclusions

Gender related work challenges affecting women scientists’ retention in the UAE and progress in STEM fields still exists. Increasing societal awareness, and endorsing governmental and institutional initiatives, policies and procedures to challenge stereotypes and promote gender equality are required.

## Author contributions

Conceptualization, A. R, L. Z, S. A, H. A, R. F, P.S; methodology, A. Z, JN, S.A; validation, A. R, J.N; analysis, A. Z, S.A, A. S, A.S.A; X.X.; investigation, X.X.; resources, X.X.; data curation, X.X.; writing—original draft preparation, S. A, A.R, J. N, A.A. writing—review and editing, all authors. All authors have read and agreed to the published version of the manuscript.

## Ethics and consent

The study was approved by the Social Sciences Ethics Committee (IRB) of the United Arab Emirates University with approval number ERSC_2022_1527 on 25/10/2022. A detailed information sheet about the purpose of the study was distributed to participants, and written informed consent was obtained from each participant before inclusion in the study. Involvement of human participants compiled with the ethical standards set forth in the Declaration of Helsinki.

## Data Availability

OSF: Factors affecting women scientists’ retention and progress STEM fields in the UAE: A cross-sectional study. https://osf.io/br983/?view_only=162d544f6536458b86c32fe62a874bb7( Almarzooqi 2024). This project contains the following underlying data:
• STEM DATASET.xlsx Data are available under the terms of the Creative Commons Attribution 4.0 International license (CC-BY 4.0). OSF: Factors affecting women scientists’ retention and progress STEM fields in the UAE: A cross-sectional study. https://osf.io/br983/?view_only=162d544f6536458b86c32fe62a874bb7( Almarzooqi 2024). This project contains the following extended data:
• Extended data 1 _STEM Survey.pdf • Extended data 2_ STROBE_checklist_cross-sectional_ATR_STEM.docx Data are available under the terms of the Creative Commons Attribution 4.0 International license (CC-BY 4.0).
