# Supplementary material for: Factors affecting women scientists’ retention and progress in STEM fields in the UAE: A cross-sectional study
Source: F1000Res. 2026 Jan 21;13:1539. Originally published 2024 Dec 20. [Version 3] doi: 10.12688/f1000research.155420.3 (PMC12775668; doi:10.12688/f1000research.155420.3)
Supplement: Peer review history

# Reviewer report: Reviewer response for version 3

**DOI:** 10.5256/f1000research.193455.r451859

**Published:** 2026-01-29

**Authors:** Patricia Vázquez-Villegas

**Affiliations:** 1 Tecnologico de Monterrey, Nuevo León, Mexico

The authors took all my comments into account.

Is the work clearly and accurately presented and does it cite the current literature?

Partly

If applicable, is the statistical analysis and its interpretation appropriate?

I cannot comment. A qualified statistician is required.

Are all the source data underlying the results available to ensure full reproducibility?

Partly

Is the study design appropriate and is the work technically sound?

Partly

Are the conclusions drawn adequately supported by the results?

Partly

Are sufficient details of methods and analysis provided to allow replication by others?

Partly

Reviewer Expertise:

Engineering Education

I confirm that I have read this submission and believe that I have an appropriate level of expertise to confirm that it is of an acceptable scientific standard.

# Reviewer report: Reviewer response for version 2

**DOI:** 10.5256/f1000research.188356.r439455

**Published:** 2026-01-06

**Authors:** Maria F Vieira

**Affiliations:** 1 University of South Australia, Adelaide, South Australia, Australia

The topic is timely, policy-relevant, and well aligned with global discussions on gender equity in STEM. While the manuscript has improved substantially from version 1, it still falls short of its potential due to limitations in framing, methodological clarity, and analytical depth. Overall, the paper is acceptable, but not yet compelling.

 Sample composition and representativeness

 The findings are interpreted broadly as representative of “STEM in the UAE,” yet the sample predominantly reflects senior, academic STEM environments. I recommend narrowing the scope of the claims more explicitly to academic and research-intensive STEM settings. In addition, sample-related limitations should be moved from a general paragraph into a clearly labeled **Study limitations** subsection to improve transparency and coherence.

 Clarify methods

 A short methodological clarification paragraph is needed to explicitly address:

- the calculated target sample size versus the achieved sample size,
- where the full survey instrument can be accessed (e.g., appendix or extended data), and
- how internal consistency was assessed across mixed item types.

In particular, the rationale for combining Likert-scale, binary, and categorical items in Cronbach’s alpha calculations remains insufficiently justified and should be clarified.

 Results are descriptive but under-interpreted

 Much of the Results section closely mirrors the figures and tables, with limited analytical interpretation. While Figures 2–7 are well designed, the accompanying text primarily describes *what* differs rather than *why* those differences matter. I suggest emphasizing effect sizes and key contrasts rather than percentages alone, and reducing redundancy between text and figures. Some figures could be merged or moved to supplementary materials.

 As a specific example, adding the frequency of theme occurrences to Table 4 would strengthen the link between the qualitative findings and the Discussion, and would better support recommendations regarding the most impactful interventions.

 Contribution undersold in the Discussion and Conclusion

 The Discussion and Conclusion would benefit from a clearer articulation of the study’s contribution. Specifically, the authors should state more explicitly:

- what this study adds beyond confirming well-documented gender biases in STEM, and
- why the UAE context provides analytically meaningful insights (e.g., its multicultural workforce and policy environment).

Minor amendments

- Define “UAE” as *United Arab Emirates* at first mention.
- Clearly state the research question(s).
- On page 5, remove the paragraph immediately preceding Table 1, as this information is repeated on page 6.
- In Table 4, clarify whether the supporting quotes are illustrative examples or verbatim participant quotes. If the latter, anonymized identifiers (e.g., Participant 1, Participant 2) should be included.
- Several figures lack fully self-contained captions (e.g., gender color coding in Figure 4; scope and interpretation in Figure 7).
- Some statements in the Discussion refer to “research has shown” without immediate citations.
- Minor grammatical and typographical errors remain and should be corrected.

Is the work clearly and accurately presented and does it cite the current literature?

Partly

If applicable, is the statistical analysis and its interpretation appropriate?

I cannot comment. A qualified statistician is required.

Are all the source data underlying the results available to ensure full reproducibility?

Yes

Is the study design appropriate and is the work technically sound?

Partly

Are the conclusions drawn adequately supported by the results?

Yes

Are sufficient details of methods and analysis provided to allow replication by others?

Partly

Reviewer Expertise:

Gender equity in STEM

I confirm that I have read this submission and believe that I have an appropriate level of expertise to confirm that it is of an acceptable scientific standard, however I have significant reservations, as outlined above.

# Reviewer report: Reviewer response for version 2

**DOI:** 10.5256/f1000research.188356.r425643

**Published:** 2025-11-28

**Authors:** Patricia Vázquez-Villegas

**Affiliations:** 1 Tecnologico de Monterrey, Nuevo León, Mexico

I thank the authors for considering my suggestions to improve their document. Now it seems more equilibrated. However, I have some more comments for you. 

 1.    I believe that now that they have conducted a qualitative analysis, they can improve their abstract to reflect the new results.  

 2.    I think there is a missing line before this “- see extended data 1) was divided into three sections: demographic, attitudes questions, and the Andrews and Withey job”

 3.    Consider revising the figures and table captions and write self-explanatory names for the Figures and tables. For example, “Figure 7. Support needed by females and males to thrive in STEM.” The figure also shows male responses, but the title does not clearly explain what we can see. 

 4.    In Fig. 4, for example, one cannot tell whether the blue color is for males or females. 

 5.    Was Fig. 1 answered only by women? What about males? What are the most influential factors for them to pursue a STEM career?

 6.    Please include a reference to the WHO calculator for cross-sectional studies, the equation, or the calculation result. Integrate the “Sample size calculation” into the Study population section. 

 7.    Could you please indicate in the text where the questionnaire is? Is it in an Appendix?

Is the work clearly and accurately presented and does it cite the current literature?

Partly

If applicable, is the statistical analysis and its interpretation appropriate?

I cannot comment. A qualified statistician is required.

Are all the source data underlying the results available to ensure full reproducibility?

Partly

Is the study design appropriate and is the work technically sound?

Partly

Are the conclusions drawn adequately supported by the results?

Partly

Are sufficient details of methods and analysis provided to allow replication by others?

Partly

Reviewer Expertise:

Engineering Education

I confirm that I have read this submission and believe that I have an appropriate level of expertise to confirm that it is of an acceptable scientific standard, however I have significant reservations, as outlined above.

# Response

**Published:** 2025-12-11

**Authors:** Saeeda Almarzooqi

**Affiliations:** Pathology, United Arab Emirates University, Al Ain, Abu Dhabi, United Arab Emirates

Dear Reviewer,

 Thank you very much for your constructive and thoughtful feedback on Version 2 of our manuscript. We appreciate the time you have taken to review the revisions, and we are pleased to address the remaining points you have highlighted.

**Q1**.I believe that now that they have conducted a qualitative analysis, they can improve their abstract to reflect the new results.

**Response:** The abstract has been revised to incorporate key findings from the qualitative analysis, mentioning the identified outcomes and participant recommendations.

**Q2.** I think there is a missing line before this “- see extended data 1) was divided into three sections: demographic, attitude questions, and the Andrews and Withey job.”

**Response:** Thank you for spotting the error. The sentence structure in the Methods section has been corrected for clarity and flow.

**Q3. **Consider revising the figures and table captions, and write self-explanatory names for the Figures and tables. For example, “Figure 7. Support needed by females and males to thrive in STEM.” The figure also shows male responses, but the title does not clearly explain what we can see. 

**Response:** Figure and table captions have been revised wherever needed to provide clear, descriptive titles that explicitly state what data is presented and for which groups.

**Q4. **In Fig. 4, for example, one cannot tell whether the blue color is for males or females. 

**Response:** We apologize for this lack of clarity. The figure has been updated to include a definitive legend within the graph itself, clearly labeling which color represents male and which represents female respondents.

**Q 5. **Was Fig. 1 answered only by women? What about males? What are the most influential factors for them to pursue a STEM career?

**Response:** The reviewer is correct. Figure 1 was based on a filter applied only to female respondents, as described in the accompanying text. For clarity and comparison, the figure has been updated, including male participation. The text was also updated, including the new data from male participants.

**Q 6. **Please include a reference to the WHO calculator for cross-sectional studies, the equation, or the calculation result. Integrate the “Sample size calculation” into the Study population section. 

**Response:** A citation for the WHO tool has been added. Integrated the “Sample size calculation” into the Study population section.

**Q7. **Could you please indicate in the text where the questionnaire is? Is it in an Appendix?

**Response:** The location is now explicitly stated in the text: "The full questionnaire is provided as Extended Data 1."

 Once again, we sincerely appreciate your valuable suggestions. We believe these revisions have further strengthened the manuscript, and we are grateful for your guidance throughout the review process.

 Kind regards,

 Prof Saeeda AlMarzooqi

# Reviewer report: Reviewer response for version 2

**DOI:** 10.5256/f1000research.188356.r425642

**Published:** 2025-11-21

**Authors:** Merryn McKinnon

**Affiliations:** 1 Australian National University, Canberra, Australia

Thank you to the authors for their thoughtful responses to suggested amendments. These have mostly been addressed. I am happy for the paper to be indexed however would request the authors do make some minor adjustments.

 Table 1 - the clarifying note of what constitutes 'other Asians' as a respondent nationality has been added underneath the table, which is appreciated, however it hasn't actually been 'linked' to anything in the table. I would recommend an asterisk or similar at the category which is then included with the descriptor e.g. Other Asians* and then under the table, *Includes participants...

 The paragraph prior to the conclusions still contains sentences stating "research has shown..." without any references included. This has not been addressed. 

 Some minor spelling/grammar and typographical errors appear.

 Finally, the authors may wish to reiterate in the conclusion the universality of their findings and the need to address the embedded bias in the structures, systems and norms of STEM worldwide.

Is the work clearly and accurately presented and does it cite the current literature?

Yes

If applicable, is the statistical analysis and its interpretation appropriate?

I cannot comment. A qualified statistician is required.

Are all the source data underlying the results available to ensure full reproducibility?

Yes

Is the study design appropriate and is the work technically sound?

Yes

Are the conclusions drawn adequately supported by the results?

Partly

Are sufficient details of methods and analysis provided to allow replication by others?

Partly

Reviewer Expertise:

gender in STEM

I confirm that I have read this submission and believe that I have an appropriate level of expertise to confirm that it is of an acceptable scientific standard.

# Response

**Published:** 2025-12-11

**Authors:** Saeeda Almarzooqi

**Affiliations:** Pathology, United Arab Emirates University, Al Ain, Abu Dhabi, United Arab Emirates

Dear Reviewer,

 Thank you very much for your constructive and thoughtful feedback on Version 2 of our manuscript. We appreciate the time you have taken to review the revisions, and we are pleased to address the remaining points you have highlighted.

  

1. **Table 1 – Clarifying Note for “Other Asians.”**

We have now incorporated an asterisk directly within the table to clearly link the category “Other Asians*” with the explanatory note. The corresponding footnote has been placed directly beneath the table and now reads:

 “*Other Asians refers to participants from various Asian countries, excluding UAE nationals”.

  

1. **Paragraph before the Conclusion – Missing References**

Thank you for pointing out the unreferenced statements. We have revised this paragraph by adding an appropriate citation for the statement beginning with “research has shown…”,

  

1. **Spelling, Grammar, and Typographical Corrections**

We have thoroughly rechecked the manuscript and corrected all minor spelling, grammar, and typographical errors.

  

1. **Conclusion – Reinforcing Universality of Findings and Addressing Structural Bias**

As suggested, we have strengthened the conclusion by adding the following sentences-to address the global relevance and universality of the findings, and

 systemic and structural biases embedded within STEM environments worldwide.

 “Although the scope of this study is limited to the UAE, the identified bias patterns and structural restrictions are consistent with those observed internationally. Addressing these concerns requires working together globally so that the deep-rooted biases found in STEM systems and practices can be removed”.

 Once again, we sincerely appreciate your valuable suggestions. We believe these revisions have further strengthened the manuscript, and we are grateful for your guidance throughout the review process.

 Kind regards,

 Prof Saeeda AlMarzooqi

# Reviewer report: Reviewer response for version 1

**DOI:** 10.5256/f1000research.170598.r354235

**Published:** 2025-08-21

**Authors:** Merryn McKinnon

**Affiliations:** 1 Australian National University, Canberra, Australia

This paper outlines a study exploring the factors affecting the retention and progression of women in STEM careers in the UAE using a cross-sectional survey.

 The context provided is useful however would benefit from a little more specificity. The statistics about the number of women graduating in STEM fields in the UAE provides part of the picture. What is the employment rate for women in computer science and engineering in the UAE? Globally these fields tend to have much lower proportions - how does the UAE compare to other countries? (It may be doing better!). Many years ago (circa 2010), in at least one UAE university, health and science were only offered to women while men were expected to study business, HR and finance. Is this split consistent across all institutions and did it still apply in 2022? The lit review also doesn't provide any background to what factors will be asked about or a brief overview of the barriers to retention and progression already identified in myriad studies. This comes in the discussion, which is arguably a bit too late.

 There is also a unique opportunity in this study in that it offers a perspective from many different nationalities. The multicultural nature of the Emirati society and workforce is not something that would be necessarily known by readers. It provides an even more interesting context within which to discuss the results so it should be covered - even briefly - here. 

 Results - figures 2 and 3 could be combined as could figs 8 and 9. It isn't clear in figs 8 and 9 if respondents feel they already have these and this is what they think is important OR if these are elements that respondents are missing. 

 Discussion - a lot of the elements discussed here likely should have been raised in the literature review. The cited junior biomedical researchers study - is that based in the UAE or elsewhere? 

 The final paragraph before the conclusion talks about "research has shown" but doesn't cite any - it should. What this paper doesn't discuss, and arguably should, is the universality of these results. These are people from a range of different cultural backgrounds working within the UAE. Each respondent brings their own cultural norms to their work, which has its own cultural norms. And the findings here resonate with studies from around the world. There is something about gender in STEM that transcends culture and geography. This paper shows this in microcosm but doesn't make it prominent. It should. 

 Minor edits - fifth para - some references need to replace numbers (6 and 9?)

 Para 6 - 'toolkit developed by some investigators' - is this still referring to the Casad et al paper or others? Please clarify/cite accordingly.

 Table 1 - 'other asians' could be made more specific as could the description of 'other' employers.

Is the work clearly and accurately presented and does it cite the current literature?

Yes

If applicable, is the statistical analysis and its interpretation appropriate?

I cannot comment. A qualified statistician is required.

Are all the source data underlying the results available to ensure full reproducibility?

Yes

Is the study design appropriate and is the work technically sound?

Yes

Are the conclusions drawn adequately supported by the results?

Partly

Are sufficient details of methods and analysis provided to allow replication by others?

Partly

Reviewer Expertise:

gender in STEM

I confirm that I have read this submission and believe that I have an appropriate level of expertise to confirm that it is of an acceptable scientific standard, however I have significant reservations, as outlined above.

# Response

**Published:** 2025-09-17

**Authors:** Saeeda Almarzooqi

**Affiliations:** Pathology, United Arab Emirates University, Al Ain, Abu Dhabi, United Arab Emirates

Approved With Reservations

 This paper outlines a study exploring the factors affecting the retention and progression of women in STEM careers in the UAE using a cross-sectional survey.

 The context provided is useful, however would benefit from a little more specificity. The statistics about the number of women graduating in STEM fields in the UAE provides part of the picture.

 What is the employment rate for women in computer science and engineering in the UAE? Globally these fields tend to have much lower proportions - how does the UAE compare to other countries? (It may be doing better!). Many years ago (circa 2010), in at least one UAE university, health and science were only offered to women while men were expected to study business, HR and finance.

 Is this split consistent across all institutions and did it still apply in 2022? The lit review also doesn't provide any background to what factors will be asked about or a brief overview of the barriers to retention and progression already identified in myriad studies. This comes in the discussion, which is arguably a bit too late.

 There is also a unique opportunity in this study in that it offers a perspective from many different nationalities. The multicultural nature of the Emirati society and workforce is not something that would be necessarily known by readers. It provides an even more interesting context within which to discuss the results so it should be covered - even briefly - here. 

 Results - figures 2 and 3 could be combined as could figs 8 and 9. It isn't clear in figs 8 and 9 if respondents feel they already have these and this is what they think is important OR if these are elements that respondents are missing. 

 Discussion - a lot of the elements discussed here likely should have been raised in the literature review.

**Response**: We have now integrated the summary of key findings in the revised Introduction/literature review section of the manuscript.

 The cited junior biomedical researchers study - is that based in the UAE or elsewhere?

**Response:** The study was conducted in the United States, and used the data from two New England biomedical research programs administered by the Medical Foundation Division of Health Resources in Action. We have now clarified the setting (United States) of the study in the revised manuscript.

 The final paragraph before the conclusion talks about "research has shown" but doesn't cite any - it should.

**Response:** We have now provided the relevant citations to the statements in the revised manuscript.

 What this paper doesn't discuss, and arguably should, is the universality of these results. These are people from a range of different cultural backgrounds working within the UAE. Each respondent brings their own cultural norms to their work, which has its own cultural norms. And the findings here resonate with studies from around the world. There is something about gender in STEM that transcends culture and geography. This paper shows this in microcosm but doesn't make it prominent. It should. 

 Response: We have now added a paragraph in the Discussion section addressing this point. We discuss how our results, derived from a uniquely multicultural context in the UAE, align with and reinforce findings from Western, Eastern, and other cultural settings. This new text argues that this convergence of evidence suggests a pervasive, trans-cultural pattern of gender bias in STEM, potentially pointing to deeper, field-specific mechanisms. We believe this revision significantly strengthens the paper's contribution and broadens its impact, and we are grateful for the suggestion.

 Minor edits - fifth para - some references need to replace numbers (6 and 9?)

 Para 6 - 'toolkit developed by some investigators' - is this still referring to the Casad et al paper or others? Please clarify/cite accordingly.

 Table 1 - 'other asians' could be made more specific as could the description of 'other' employers.

- **Is the work clearly and accurately presented and does it cite the current literature?**

Yes

- **Is the study design appropriate and is the work technically sound?**

Yes

- **Are sufficient details of methods and analysis provided to allow replication by others?**

Partly

- **If applicable, is the statistical analysis and its interpretation appropriate?**

I cannot comment. A qualified statistician is required.

- **Are all the source data underlying the results available to ensure full reproducibility?**

Yes

- **Are the conclusions drawn adequately supported by the results?**

Partly

# Reviewer report: Reviewer response for version 1

**DOI:** 10.5256/f1000research.170598.r369763

**Published:** 2025-03-21

**Authors:** Patricia Vázquez-Villegas

**Affiliations:** 1 Tecnologico de Monterrey, Nuevo León, Mexico

The study demonstrates an interesting approach to obtaining information regarding the challenges faced by female graduates in STEM fields in the UAE. The authors conducted a cross-sectional study by distributing surveys using the snowball sampling method. They collected 165 valid responses on the challenges individuals face in staying in STEM fields. 62% of the respondents were men. I propose that the article's authors consider my following comments, if they consider it relevant:

 1. My biggest concern is: If the study focuses on women, why not wait until the expected number of female responses was collected? I do not disregard the fact that men also face workplace inequities in STEM. However, would it be worthwhile to frame the study as a comparative analysis between men and women? Such an approach could generate insights into addressing diversity and finding common ground to eliminate inequities.

 2. On page 3, it is mentioned: "(consisting of 28 open and closed-ended questions - see extended data 1)." Please report exactly how many were open-ended and how many were closed-ended.

 3. If the surveys contained open-ended questions, why were the responses not analyzed using thematic analysis or coding? For example, Question 22 asks about perceived barriers, yet these are not described in the main manuscript, nor are Questions 43 and 44.

 4. How was Cronbach’s alpha calculated for questions of different types? For instance, Question 2 follows a Likert scale, while others are Yes/No, multiple-choice, or true/false. I believe the authors should better explain how the survey results were analyzed.

 5. It is necessary to present information about the number of questions eliminated during the pilot phase.

 6. The sample size calculation is presented, but the result is not stated. Is it 165? Did the sampling stop at participant 165?

 7. Some acronyms, such as MIT or WHO, need to be defined. Ensure that all acronyms are introduced with their full form when first mentioned.

 8. Can the authors identify relationships between respondents’ characteristics and their answers? For example, if a participant is a woman and also has children, does this intensify the challenges they face?

 9. I am unsure whether the phrase "bread-earners" is appropriate for this manuscript.

 10. Table 2 should be explained in more detail. Explicitly describe what the statistical test results mean.

 11. Most of the text in the results section repeats what is already observed in the figures and tables. While this is not entirely incorrect, it might be better to explain more about the meaning or implications of these results. Otherwise, I suggest merging some figures into one (placing them side by side) to save space and improve readability, for example, Figures 8 and 9.

 12. The authors' conclusion is very limited. For instance, it is a fact that, in many parts of the world, women still face challenges in STEM fields. But what does this new study suggest to counteract the already well-known systemic challenges? What is the main contribution of this work? Please, highlight the significance of this work.

 13. Please, add titles to the subsections, such as "Study Limitations." I also suggest adding subtitles in the discussion section, to maked more organized.

 Based on the concerns raised in the previous comments, here are some suggestions to accomplish the questionaire of the peer-review form:

 1. Current literature? → Partly

 Authors may expand the literature review to better contextualize findings within existing and recent (not more than 5 years old) research on gender disparities in STEM, particularly in the UAE and similar regions.

 2. Appropriate desig: → Partly

 Please, justify why qualitative analysis was not used. If feasible, include some qualitative analysis to capture nuanced insights from open-ended responses. Also, clarify why the mixed-gender approach was necessary or consider conducting a gender-comparative analysis to provide additional insights.

 3. Sufficient details of methods → Partly

 Authors can clearly describe how the internal consistency was measured across diverse question types and justify the approach taken. Also, provide the actual calculated sample size and explain whether data collection stopped at 165 participants or if any adjustments were made.

 4. Appropriateness of statistical analysis  → I would say partly

 Please, expand the description of Table 2, detailing the significance of the results and how they support the study’s conclusions. Provide rationale for the selection of the statistical methods.

 5. Reproducibility → Partly

 Authors may include information about how many questions were eliminated and why, as this is important for reproducibility. If possible, provide a supplementary file with anonymized responses for open uestions or summaries of key themes.

 6. Adequate conclusions → Partly

 Discuss potential interventions or policy recommendations based on the findings. Authors may address how organizations or policymakers could use these insights to mitigate gender-based challenges in STEM.

 I hope these comments are useful to you!

Is the work clearly and accurately presented and does it cite the current literature?

Partly

If applicable, is the statistical analysis and its interpretation appropriate?

I cannot comment. A qualified statistician is required.

Are all the source data underlying the results available to ensure full reproducibility?

Partly

Is the study design appropriate and is the work technically sound?

Partly

Are the conclusions drawn adequately supported by the results?

Partly

Are sufficient details of methods and analysis provided to allow replication by others?

Partly

Reviewer Expertise:

STEM education, education for sustainability, continuing education.

I confirm that I have read this submission and believe that I have an appropriate level of expertise to confirm that it is of an acceptable scientific standard, however I have significant reservations, as outlined above.

# Response

**Published:** 2025-05-22

**Authors:** Saeeda Almarzooqi

**Affiliations:** Pathology, United Arab Emirates University, Al Ain, Abu Dhabi, United Arab Emirates

**Reviewer 1**

 The study demonstrates an interesting approach to obtaining information regarding the challenges faced by female graduates in STEM fields in the UAE. The authors conducted a cross-sectional study by distributing surveys using the snowball sampling method. They collected 165 valid responses on the challenges individuals face in staying in STEM fields. 62% of the respondents were men. I propose that the article's authors consider my following comments, if they consider it relevant:

**1. My biggest concern is: If the study focuses on women, why not wait until the expected number of female responses was collected? I do not disregard the fact that men also face workplace inequities in STEM. However, would it be worthwhile to frame the study as a comparative analysis between men and women? Such an approach could generate insights into addressing diversity and finding common ground to eliminate inequities.**

 Thank you for this thoughtful comment and valuable suggestion. Our original intent was indeed to focus primarily on women’s retention and progression in STEM fields and also understand issue from both gender view. However, during the data collection with the snowball sampling strategy, a significant proportion of responses came from male participants working in the same fields and institutions. Rather than excluding these responses, we decided to include them to enrich the analysis and allow for comparative insights between genders.

 We acknowledge the limitation regarding the smaller proportion of female respondents (38%) and have now elaborated on this issue in the **Limitations** section of the revised manuscript.

 Additionally, we agree with the reviewer that the comparative perspective strengthens the study by shedding light on the differing experiences of men and women in STEM, which may help in identifying shared and divergent challenges. Therefore, we have reframed parts of the analysis and discussion to reflect this comparative lens more clearly.

**2. On page 3, it is mentioned: "(consisting of 28 open and closed-ended questions - see extended data 1)." Please report exactly how many were open-ended and how many were closed-ended.**

 Thank you very much for highlighting this point. Upon reviewing the final version of the survey tool, we realized that the description in the manuscript was outdated and inaccurately stated the number of items.

 The survey actually consisted of **44 questions**, not 28 as originally mentioned. Of these:

- **6 were open-ended questions**, which invited participants to share their personal experiences, insights, or suggestions (e.g., questions 3, 11, 14, 22, 43, and 44).
- **38 were closed-ended questions**, covering a range of formats including Likert scales, multiple-choice, true/false, and rating scale items.

We have corrected this information in the revised manuscript to ensure full transparency and accuracy in describing the methodology. We truly appreciate your attention to detail—it helped us improve the clarity and completeness of the manuscript.

**3. If the surveys contained open-ended questions, why were the responses not analyzed using thematic analysis or coding? For example, Question 22 asks about perceived barriers, yet these are not described in the main manuscript, nor are Questions 43 and 44.**

 Thank you for this excellent observation. You are absolutely right that analyzing the open-ended responses—particularly for Question 22—would add valuable depth and context to our findings.

 Initially, our focus was on presenting the quantitative results of the study, and we did not include a formal thematic analysis of the qualitative items due to space constraints and our emphasis on the statistical findings. However, we fully agree that the rich narratives provided by respondents offer important insights into the lived experiences of women in STEM.

 In response to your suggestion, we have now conducted a thematic analysis of the responses to Question 22. The open-ended responses were independently reviewed and coded by two researchers using an inductive approach. Five main themes emerged:

 Themes from Reported Barriers to Women's Advancement in STEM (Q22 Open-Ended Responses)

 Theme

 Definition

 Supporting Quotes

 1. Gender Bias in Hiring, Pay, and Promotion

 Systemic discrimination where women receive unequal treatment in hiring, compensation, promotion, or leadership access.

 "His salary was higher", HR replied that he is a man, he has to support the family."

 "Men promoted or given responsibility when CV of the female employee is clearly superior"

 2. Motherhood and Family Responsibilities as Career Barriers

 Women's roles as mothers and caregivers negatively affect their career progress or continuity.

 "Female pharmacists, might request later maternity leave."‌

 "Career gaps due to childbearing" do not affect men."‌

 3. Lack of Institutional Support and Flexibility

 Inadequate policies or accommodations for women's needs, such as flexible hours, childcare, or post-maternity reintegration.

 "Inflexible workloads, giving women more opportunities to have part-time hours, online meetings, etc."‌

 "Lack of support after maternity leave."‌

 4. Cultural and Social Norms Reinforcing Gender Roles

 Societal and workplace norms that reinforce traditional female roles and diminish professional credibility or opportunities.

 "Women are questioned about their commitment if they want both a family and a career."‌

 "Cultural need for women to manage household and childcare."‌

 5. Exclusion from Networks, Resources, and Fair Processes

 Limited access to support, networks, or fair processes due to male-dominated environments, favoritism, or underrepresentation.

 "Female faculty did not receive lab space,male did."‌

 "Not a single woman in university management."‌

 Thematic Analysis - Women Leaving Science Careers Early ( Q43 Open-Ended Responses)

 Theme

 Definition

 Supporting Quotes

 1. Family Responsibilities and Caregiving Burden

 The overwhelming burden of caregiving duties (e.g., children, elderly, spouse) often forces women to prioritize home over career.

 "Family commitments. Family responsibilities. Care for children and the elderly."

 2. Lack of Work-Life Balance and Flexibility

 Rigid work hours, long shifts, and inability to adjust roles to accommodate personal life lead to burnout and early career exit.

 "Inflexibility for work time. Workload. Long hours and heavy work responsibilities."

 3. Poor Workplace Culture and Mental Health Strain

 Toxic work environments, stress, mental exhaustion, and lack of appreciation contribute to resignation.

 "Poor work environmentâ€¦ they choose mental health over money and career advancement."

 4. Gender Discrimination and Lack of Career Progression

 Inequity in promotions, lack of recognition, and systemic bias demoralize women and block advancement.

 "Lack of promotion. Working hard but suppressed by male colleagues. Bias and discrimination."

 5. Structural and Policy Gaps (e.g., parental leave, support systems)

 Lack of institutional support such as maternity leave, reintegration policies, and job security for women scientists.

 "Insufficient parental leave policies. Difficult to relaunch career after maternity. Lack of stable positions."

 When asked about their recommendations to the employer and government on

 how to improve the employment and career environment for women scientists, respondents recommended improving the work environment for women scientists by introducing flexible working hours, remote work options, and part-time roles to support work-life balance. They emphasized the need for better childcare support, including on-site daycare and extended paid maternity leave, along with policies that facilitate reintegration after childbirth. Calls were also made for equitable hiring, promotion, and leadership opportunities, more mentorship and female role models, and increased recognition through fair pay and dedicated research funding. Institutional reforms, such as clear gender equality policies and accountability measures, were seen as essential.

 These themes have now been incorporated into the revised results and discussion sections of the manuscript. We are grateful for your feedback, which helped us enhance the richness of our findings.

**4. How was Cronbach’s alpha calculated for questions of different types? For instance, Question 2 follows a Likert scale, while others are Yes/No, multiple-choice, or true/false. I believe the authors should better explain how the survey results were analyzed.**

 We thank the reviewer for this comment. We agree that multiple-choice, or true/false questions are not applicable in the reliability analysis carried out using Cronbach’s alpha.

 In this analysis, a reliability analysis was carried out on three subscales namely: the attitude, satisfaction, retention subscales using Cronbach’s alpha (α). Overall, Cronbach’s alpha for the three subscales indicated the questionnaire reached good reliability (α = 0.88). The following text has been added now to the manuscript [Pages: XX-XX: Lines: XX-XX]

 A reliability analysis was carried out on three subscales namely: the attitude, satisfaction, retention subscales using Cronbach’s alpha (α). Overall, Cronbach’s alpha for the three subscales indicated the questionnaire reached good reliability (α = 0.88). In details, the attitude subscale was found to have questionable reliability (9 items (Q23.1 to Q23.9); α = .63), the satisfaction subscale indicated acceptable reliability (8 items (Q28 to Q35); α = .78), and the retention subscale reached excellent reliability (7 items (Q36 to Q41); α = .92). Most items appeared to be worthy of retention, resulting in a decrease in the alpha if deleted from the subscale. The one exception to this was item Q23.1 in attitude subscale “ *It is important to take action to increase the involvement of women and girls in STEM fields.*”, which would increase the alpha to α = 0.73 if deleted. Therefore, removal of this item was considered. Furthermore, one question in the retention subscale “ *Please rate the importance of Paid leadership development related to your workplace (7 stars are the highest score and 1 star is the lowest score)*” was removed during pilot phase validity due to difficulty in comprehending the question by the respondents.

**5. It is necessary to present information about the number of questions eliminated during the pilot phase.**

 This question has been answered in 4 above and is reflected in the manuscript.

**6. The sample size calculation is presented, but the result is not stated. Is it 165? Did the sampling stop at participant 165?**

 Thank you for highlighting this important point. We acknowledge that the explanation around the sample size calculation and final number of participants was not fully clear in the original manuscript.

 Using the WHO sample size calculator for cross-sectional studies—with a 95% confidence level, 5% margin of error, and an assumed response distribution of 50%—the calculated sample size was 384. However, we were only able to collect 165 valid responses during the data collection period (November 2022 to July 2023).

 This shortfall was largely due to the sensitive nature of the topic, which likely impacted participation rates, particularly among female professionals in STEM fields. We fully recognize this as a limitation and have now explicitly stated both the calculated sample size and the final number of responses in the revised manuscript. We have also expanded the limitations section to reflect how this may affect the generalizability of the findings.

 We appreciate your thoughtful feedback, which helped us improve the transparency and completeness of our methods reporting.

 Below is sentence added to the limitation:

 “The study did not reach the initially calculated sample size, primarily due to the sensitivity of the topic, which may affect the representativeness and generalizability of the findings.”

**7. Some acronyms, such as MIT or WHO, need to be defined. Ensure that all acronyms are introduced with their full form when first mentioned.**

 In response to your comment, we have carefully reviewed the manuscript and ensured that all acronyms—including Massachusetts Institute of Technology (MIT) and World Health Organization (WHO)—are now introduced in full upon first mention. This has been corrected throughout the revised version of the manuscript.

 We appreciate your attention to detail, which has helped us improve the readability of the paper.

**8. Can the authors identify relationships between respondents’ characteristics and their answers? For example, if a participant is a woman and also has children, does this intensify the challenges they face?**

 We agree that exploring associations between the various challenges and participants’ characteristics, such as number of children, would be valuable. However, this is not within the scope of our study, and may not be appropriate given our small to medium sample size, especially when further split across categories of participants’ characteristics. Such analysis was only performed for the main outcome variable — “experience of gender inequality” — as presented in Table 2. 

****

**9. I am unsure whether the phrase "bread-earners" is appropriate for this manuscript.**

 Suggest to change to economic provider or principal financial supporter.

**10. Table 2 should be explained in more detail. Explicitly describe what the statistical test results mean.**

 We thank you the reviewer for this valuable comment. The narrative for the results presented in Table 2 has been revised accordingly.

**11. Most of the text in the results section repeats what is already observed in the figures and tables. While this is not entirely incorrect, it might be better to explain more about the meaning or implications of these results. Otherwise, I suggest merging some figures into one (placing them side by side) to save space and improve readability, for example, Figures 8 and 9.**

 We agree with this valuable observation by the reviewer. Hence, we have merged Figures 8 and 9 to save space and improve readability.

**12. The authors' conclusion is very limited. For instance, it is a fact that, in many parts of the world, women still face challenges in STEM fields. But what does this new study suggest to counteract the already well-known systemic challenges? What is the main contribution of this work? Please, highlight the significance of this work.**

 The conclusion has been revised to emphasize the study’s significance, its implications for policy, and actionable interventions to address systemic barriers. To maintain conciseness, this section focuses on key takeaways, while a more detailed discussion of potential solutions including policy recommendations is provided under the "Potential Interventions & Policy Recommendations" subsection in the Discussion.

**13. Please, add titles to the subsections, such as "Study Limitations." I also suggest adding subtitles in the discussion section, to maked more organized.**

 We have added the subsections in the Discussion as suggested by the reviewer.

**Based on the concerns raised in the previous comments, here are some suggestions to accomplish the questionaire of the peer-review form:**

**1. Current literature? → Partly**

**Authors may expand the literature review to better contextualize findings within existing and recent (not more than 5 years old) research on gender disparities in STEM, particularly in the UAE and similar regions.**

 A recent study looking to challenges impacting advancing in leadership positions in higher education in the UAE, demonstrate similar challenges to women in STEM fields. It highlights the deficiencies in current policies and gender biases that hinders women’s progression in these roles.  Similarly, it illustrates the need for training and initiative to ensure work-life balance **( Reference: Al-Naqbi SA, Aderibigbe SA. Advancing Women’s Leadership in United Arab Emirates Higher Education: Perspectives from Emirati Women. *Education Sciences*. 2024; 14(9):1002)**

 The importance of initiatives that offer training in STEM and improve institutional policies to reduce gender disparity is highlighted in initiatives of University of Sharjah

**( reference : Bani-Issa W, Abu Talib M, Timmins F, Brenner M, Alameddine M. Developments in empowering and supporting women's role in scientific research in the United Arab Emirates. J Adv Nurs. 2024 Jan;80(1):4-7. doi: 10.1111/jan.15731. Epub 2023 Jun 12. PMID: 37309056.)**

**2. Appropriate design: → Partly**

**Please, justify why qualitative analysis was not used. If feasible, include some qualitative analysis to capture nuanced insights from open-ended responses. Also, clarify why the mixed-gender approach was necessary or consider conducting a gender-comparative analysis to provide additional insights.**

**3. Sufficient details of methods → Partly**

**Authors can clearly describe how the internal consistency was measured across diverse question types and justify the approach taken. Also, provide the actual calculated sample size and explain whether data collection stopped at 165 participants or if any adjustments were made.**

**4. Appropriateness of statistical analysis  → I would say partly**

**Please, expand the description of Table 2, detailing the significance of the results and how they support the study’s conclusions. Provide rationale for the selection of the statistical methods.**

 We thank you the reviewer for this valuable comment. The narrative for the results presented in Table 2 has been revised accordingly. The data analysis section has also been revised to include reason for the choice of the chosen statistical method.

**5. Reproducibility → Partly**

**Authors may include information about how many questions were eliminated and why, as this is important for reproducibility. If possible, provide a supplementary file with anonymized responses for open uestions or summaries of key themes.**

 The first part was answered in point 4 at the beginning of this document and a text has been added now to the manuscript [Pages: XX-XX: Lines: XX-XX].

**6. Adequate conclusions → Partly**

**Discuss potential interventions or policy recommendations based on the findings. Authors may address how organizations or policymakers could use these insights to mitigate gender-based challenges in STEM.**

 Based on the reviewer’s suggestion, we have revised the conclusion emphasizing the need and implementation of organizational policies to reduce the gender-based biases in STEM. Please also refer to the response to comment # 12.

**I hope these comments are useful to you!**

- **Is the work clearly and accurately presented and does it cite the current literature?**

**Partly**

- **Is the study design appropriate and is the work technically sound?**

**Partly**

- **Are sufficient details of methods and analysis provided to allow replication by others?**

**Partly**

- **If applicable, is the statistical analysis and its interpretation appropriate?**

**I cannot comment. A qualified statistician is required.**

- **Are all the source data underlying the results available to ensure full reproducibility?**

**Partly**

- **Are the conclusions drawn adequately supported by the results?**

**Partly**
